# The effects of total knee arthroplasty on knee proprioception of patients with knee osteoarthritis: a meta-analysis

**DOI:** 10.1186/s13018-022-03142-0

**Published:** 2022-05-07

**Authors:** Ya-Yue Xue, Jing-Nan Shi, Kuan Zhang, Hao-Hua Zhang, Song-Hua Yan

**Affiliations:** 1 School of Biomedical Engineering, Capital Medical University, No. 10 Xitoutiao, You An Men Wai, Fengtai District, Beijing, 100069 People’s Republic of China; 2 Beijing Key Laboratory of Fundamental Research on Biomechanics in Clinical Application, Capital Medical University, Beijing, 100069 People’s Republic of China; 3 Orthopedics Department, Beijing Jishuitan Hospital, 31 Xinjiekou East Street, Xicheng District, Beijing, 100035 People’s Republic of China

**Keywords:** Knee proprioception, Balance, Knee osteoarthritis (KOA), Total knee arthroplasty (TKA), Meta-analysis

## Abstract

**Background:**

Studies have given some pieces of evidence for the effect of total knee arthroplasty (TKA) on knee proprioception of patients with knee osteoarthritis (KOA), but their results were conflicting. This review was performed to provide an updated evidence-based meta-analysis investigating the influence of TKA on knee proprioception.

**Methods:**

The electronic databases including PubMed, Google Scholar, and the Cochrane Library were accessed from their inception to March 2020. Two reviewers identified the studies that met the selection criteria for this review. Information on study type, participants, follow-up time, and outcome measures was extracted. Methodological quality was independently assessed by two reviewers using the Cochrane Handbook 5.1.0. Eleven studies with 475 participants were included in the meta-analysis.

**Results:**

The *I*^2^ index assessed the heterogeneity between studies. The results showed that the pooled standard mean difference of mean angle of error was − 0.58° (95% CI − 1 to – 0.16; *P* = 0.007; *I*^2^ = 69%), and the joint position sense of KOA patients was better after TKA surgery than that before surgery. Pooled standard mean difference of displacement of center of pressure (COP) was − 0.39 (95% CI − 0.72 to − 0.06; *P* = 0.02; *I*^2^ = 51%), and KOA patients had better static balance after TKA surgery than before surgery.

**Conclusions:**

To conclude, no standardized comprehensive evaluation protocol presently exists though different assessment tools are available to measure proprioception. Contrasting results were found in the literature since some studies found that TKA improves proprioception in KOA patients, while others found no difference in proprioception. These differences are seen whether the proprioception was assessed by joint position sense (JPS), or it was indirectly assessed by static balance. However, the lack of sufficient data on the threshold to detect passive movement (TTDPM) and dynamic balance made it difficult to draw a conclusion about whether or not the sense of motion improved after surgery. The method for measuring and evaluating knee joint force sense is worth paying attention, which will make progress with knee proprioception on TKA patients.

## Background

Total knee arthroplasty (TKA), aiming at providing pain relief and improving physical function and the overall quality of life, is the surgical procedure considered as the gold standard, when subjects present advanced knee osteoarthritis (KOA), especially those unresponsive to pharmacological treatments [1]. However, functional impairment, gait abnormalities, and a considerable risk of falls are still present after performing total knee replacement surgery [2–4]. Patients with knee OA undergoing TKA may present further derangement of proprioception and balance control since the surgical will remove some tissues, while people always concentrated on the improvement in the symptoms and the joint function [5].

Proprioception fulfills roles in feedback and feedforward sensorimotor control and regulation of muscle stiffness. It is generally believed that proprioception includes joint position sense and joint motion sense, while some believe that the sense of force is a third key aspect [6]. Proprioception is one of the most significant factors in balance, joint stability, graceful movement, coordination, and injury prevention [7]. It is said that balance measurement is the indirect method evaluating the proprioception. In the knee, proprioception assumes three fundamental functions for the joint: stabilization during static posture, protection against excessive and possible injurious movements via reflex responses, and coordination of complex movements [8]. Proprioception involves a wide set of receptors located within joints, muscles, and tendons (e.g., Golgi tendon receptors sense changes in muscle tension). These mechanisms play a fundamental role in providing information on muscle dynamics to the central nervous system.

It is generally believed that proprioception resulted impaired in established KOA, while no difference was found between early KOA patients and age-matched control subjects, in terms of repositioning error of knee position sense [9]. However, the effects of TKA on joint proprioception of KOA patients remained contradictory. Some researchers suggested that proprioception would be reduced since the surgical removed some tissue, while the receptors might be located in these tissues. Previous studies on proprioception of TKA patients have not drawn consistent conclusions. Some authors have reported decreases [10–12], but other authors have reported positive changes in joint proprioception after TKA [13–17]. It is believed that contradictory results have been reported in the literature thus far on whether surgical knee proprioception is deficient or not post-surgery, mainly due to the use of different methods to assess knee proprioception [18]. However, decline of proprioception will lead to decreased joint stability, loss of control of joint movement, and abnormal gait. Therefore, proprioception is very important for knee function, and the recovery of proprioception is an important factor in the functional rehabilitation of knee joint.

The objective of the study was to undertake a meta-analysis investigating the effects of TKA on knee proprioception, measured using reproduction of passive positioning, threshold to detect passive movement or balance techniques.

## Methods

### Data sources

The following electronic databases were accessed from their inception to March 2020: PubMed, Google Scholar, and the Cochrane Library. Key terms were: ((total knee arthroplasty) OR (total knee replacement)) AND ((proprioception) OR (joint position sense, JPS) OR (joint motion sense) OR (sensorimotor) OR (postural control) OR (postural sway) OR (balance)). Limits of the search were: English language studies, human studies, and peer-reviewed published full access articles. Unpublished literature and trial registries of current studies were not included in the search.

### Study selection

Studies were eligible for inclusion if they (1) investigated proprioception of the knee following TKA surgery (cruciate retaining prosthesis, CR-TKA, or posterior stabilized prosthesis, PS-TKA), (2) recruited patients with TKA surgery, excluding participants those who underwent TKA surgery not for KOA, and (3) included a primary outcome measure of knee proprioception measured by mean angle of error in degrees. The primary outcome measure could take two forms: Studies measuring knee kinesthesia used the threshold to detect passive movement (TTDPM) method where the mean angle of error was defined as the difference in degrees from initiation of motion and the participant’s perception of motion, and studies measuring JPS utilizing an index angle matching method in which the mean angle of error was defined as the difference in degrees between the target angle and the angle reproduced by the participant. The type of control measure (the participant’s contralateral leg or the leg of an external matched control) was also collected along with the corresponding data.

Duplicates were removed at first. The titles and abstracts were screened, and articles which obviously did not meet the selection criteria were removed. The full text of the remaining studies was then checked against the selection criteria. Studies with outcome data that did not meet our criteria were excluded at this stage. The selection of appropriate articles was conducted by two authors.

### Quality assessment

The methodological quality of the studies that met the selection criteria was appraised by two of the researchers independently to identify studies that had a low risk of bias. Evaluation of the quality of the literature was included based on the risk bias assessment tool provided in the Cochrane Handbook 5.1.0 [19]. This handbook comprises eight potential sources of bias: consecutive or randomized sampling described, baseline characteristics of the groups are comparable, level of podiatric care provided equally between groups, completeness of outcome data, blinding of outcome assessors, bias in internal statistics, valid and reliable outcome measures, and selective outcome reporting. The scores were summarized for items on the assessment. Each item was divided into ‘L’ (low overall risk of bias that is unlikely to significantly impact the results), ‘U’ (unclear risk of bias with potential to alter results), and ‘S’ (significant risk of bias resulting in reduced confidence in results). Studies of moderate to good quality (that is 3–7 items are ‘*L*’) were selected as providing data of sufficient low risk of bias to enter into the meta-analysis.

### Data extraction and analysis

Studies that met the eligibility criteria and were of sufficient quality were included in the meta-analysis. The data extracted by this review, including the type of study, the number of participants in trial and control group, gender and age of participants, follow-up time, mean angle of error measured using TTDPM and/or JPS methods, and movement of the center of pressure (COP), were collected in a unique database to be analyzed according to the aim of our study.

The comparisons were made with an inverse variance method and presented as forest plots using Review Manager Software (version 5.4). The continuity variable was represented by weighted mean difference (WMD) or standard mean difference (SMD). The heterogeneity was tested using *I*^2^ percentages to consider the impact potential heterogeneity would have on the meta-analysis. When there was heterogeneity across studies (*P* < 0.1, *I*^2^ > 50%), random effect model was used, whereas fixed effect model was used. Sensitivity analysis was conducted to judge the stability and strength of results. Funnel plots were used to assess publication bias.

## Results

### Study selection

**Fig. 1:**
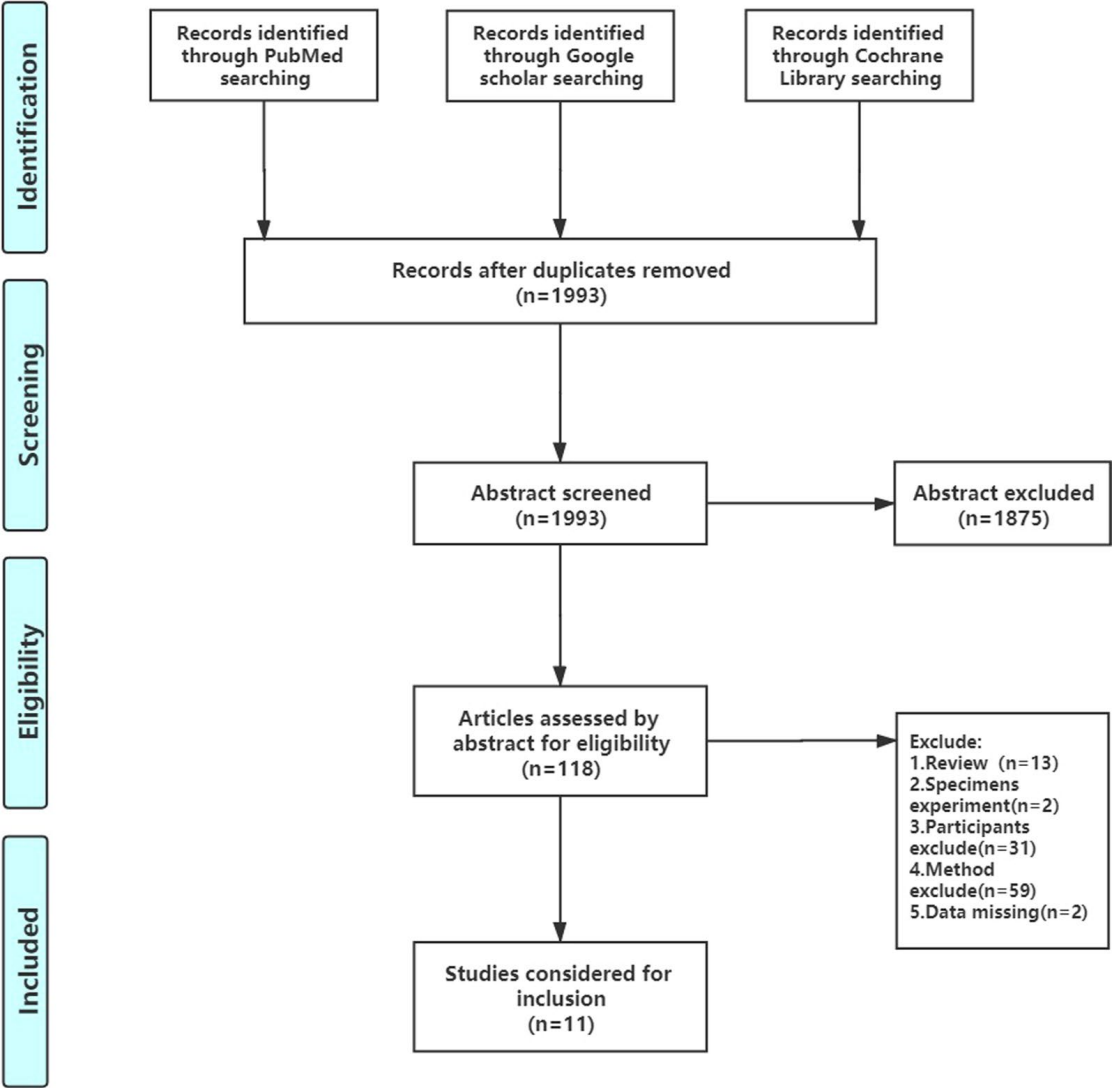
Flowchart detailing the selection process

The initial search strategy yielded 1993 articles, 1875 of which were excluded by title. Screening of the abstracts of the remaining 118 articles revealed that 13 were system review. A further 92 articles were excluded as they did not fully meet the inclusion criteria; the main exclusion factor was the use of techniques to measure proprioception other than TTDPM or JPS or balance. Two studies were excluded for data missing, and the main reasons for missing data were that median data were presented instead of mean data. Finally, eleven studies were selected in meta-analysis. The screening process of the study is shown in Fig. 1.

### Basic characteristics and quality assessment

Eleven studies involving 353 TKA patients were selected. Outcome measures included JPS, TTDPM, balance index, the path, and velocity of COP. Basic characteristics of the included studies are shown in Table 1. Among 353 TKA patients, 316 had a TKA surgery, 17 had a UKA surgery, and 20 had TKA in both knees. There were 122 healthy controls from four studies. The participants’ opposite leg was used as the control in two studies. Eight studies compared proprioception before and after surgery. Three studies compared the effects of different prosthesis types on proprioception. JPS was used to assess 172 TKA patient’s proprioception in five articles, while TTDPM was used in two studies, balance index was used in two studies, and the path of COP was used in seven studies.

**Table 1:** Basic characteristics of the included studies

Study	Participants (E/C)	Gender (M/F)		Age, mean (SD)		Follow-up time	Method of measuring proprioception	Method of measuring balance
		TKA patients	Controls	TKA patients	Controls			
Geza Pap et al. [20]	15/15	6/9	6/9	60 (56–73)	60 (56–73)	4.6 (4–6) years	TTDPM	
Wada et al. [21]	38(20CR,18PS)/23	3/35	2/21	72.6 (58–80)	71.5 (60–76)	Before and 18 (12–25) months after surgery	JPS	
Swanik et al. [16]	20(10CR,10PS)	13/7		CR 71.1 ± 6.3 PS 69.4 ± 5		6 weeks	JPS, TTDPM	Balance Index
Isaac et al. [22]	17/17(UKA)	7/10	9/8	65.8	65.5	1 day before surgery 6 months postoperatively	JPS	UST COP
Pazit Levinger et al. [23]	35	19/16		67.4 ± 7.3		Prior to the surgery 12 months following surgery	JPS	COP
Yoshinori Ishii et al. [24]	22/20 Bilateral	4/18	1/19	73 ± 5	72 ± 7	UG:preoperatively, 1 year, 2 years post-operation in BG:preoperatively, 1 day before second TKA,1 year after the second TKA		COP
Anna Słupik et al. [25]	62/74	7/55	8/66	68.8 ± 7.4	67.5 ± 6.6	1–2 days before surgery, 8 days after surgery (5–12), 100 days after the surgery (82–129)	JPS	
Cho et al. [26]	12	0/11		61.7 ± 7.3		4.5 ± 1.4 days before surgery,11.3 ± 1.0 days after surgery		Balance Index and UST COP
Stan et al. [27]	10	7/3		63.5		2 days before surgery, 7 days after surgery		COP
Vahtrik et al. [28]	40/10	0/40	0/10	60.2 ± 7.6	59.5 ± 6.6	1 day before,3 and 6 months after surgery		COP
Vandekerckhove et al. [29]	45(27CR,18PS)	CR 7/20 PS 6/12		CR 70.5 ± 6.4 PS 60.8 ± 8.4		CR: 2.9 ± 0.8 years PS: 3.1 ± 0.8 years		mCTSIB and UST

**Fig. 2:**
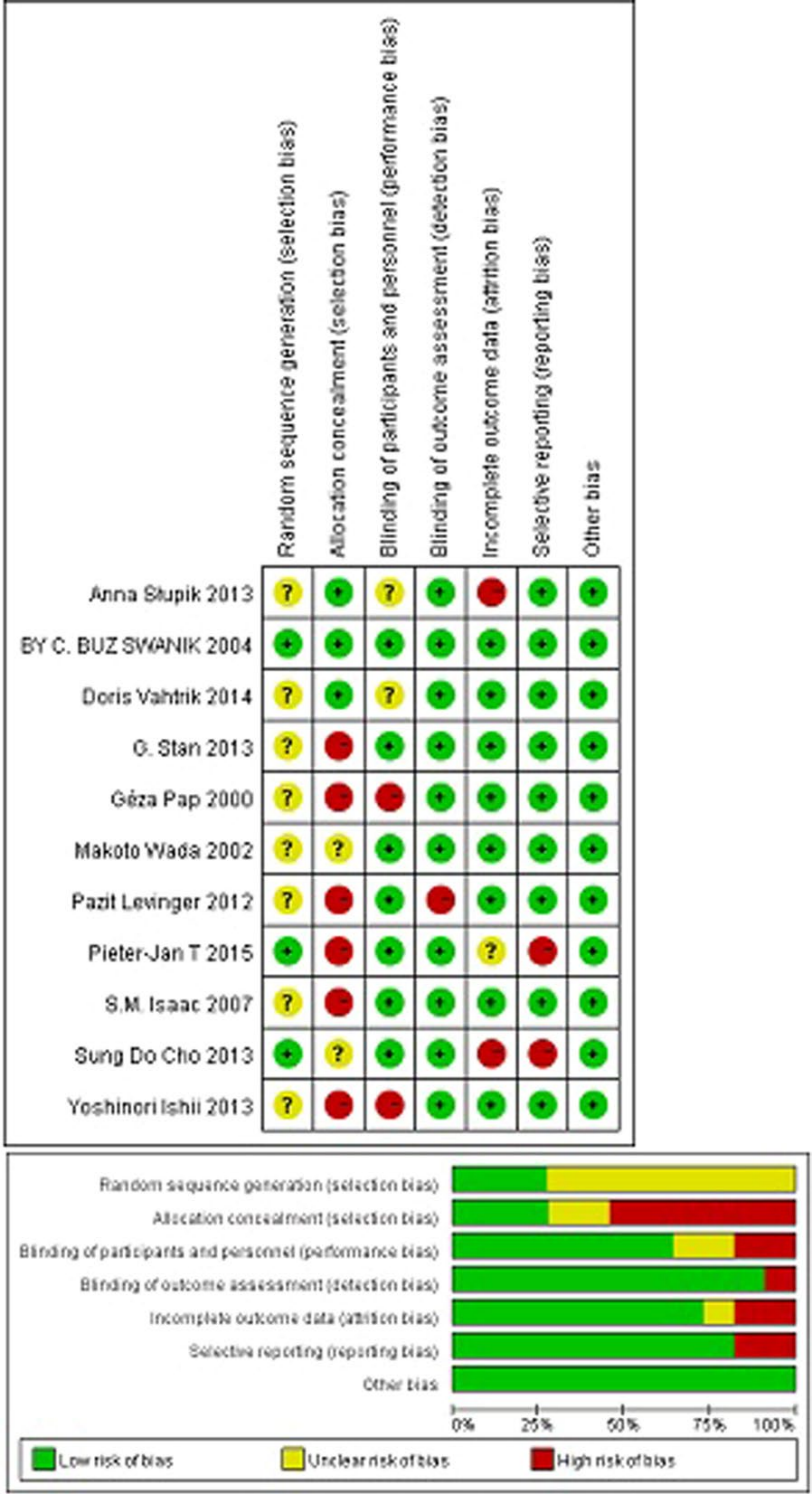
Risk of bias assessment in the included studies. Green is low risk, red is high risk, and yellow is unknown risk

Among the included papers, three mentioned random grouping. Blindness was not described in two papers. Two studies blinded assessors to the type of participant. Two studies referred to subjects lost to follow-up or excluded. Complete results were not reported in two articles. All eleven selected studies were of moderate quality. The quality assessment process is shown in Fig. 2.

## Synthesis of results

### Effects of TKA on knee proprioception

**Fig. 3:**
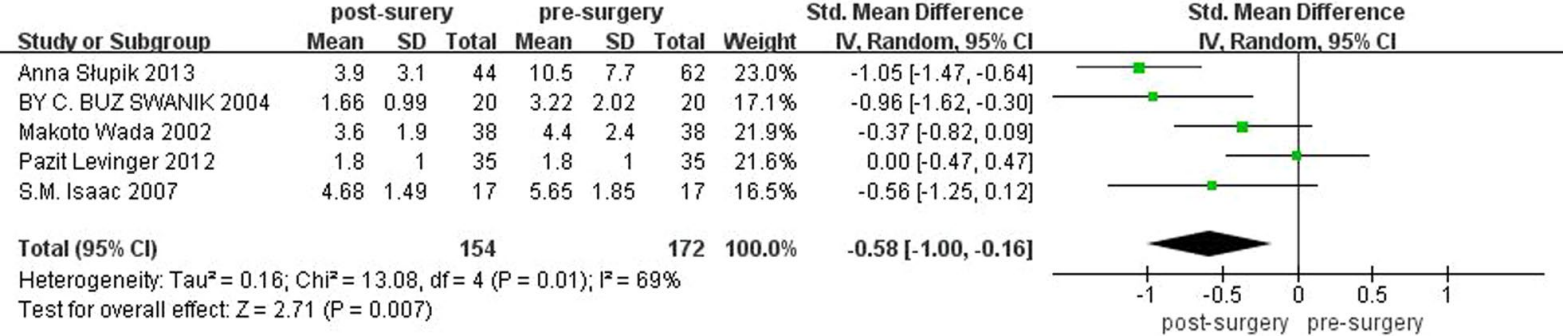
Meta-analysis results of the JPS

Five studies compared subjects' proprioception before (*n* = 172) and after (*n* = 154) surgery using the JPS. There was significant heterogeneity across the studies, so random effects model analysis was used in this part. The pooled standard mean difference of mean angle of error was − 0.58° (95% CI − 1 to − 0.16; *P* = 0.007; *I*^2^ = 69%). The results of JPS are shown in Fig. 3. It indicated that in KOA patients, the mean angle of error after operation was lower than that before operation, and the joint position sense was better after TKA surgery.

**Fig. 4:**
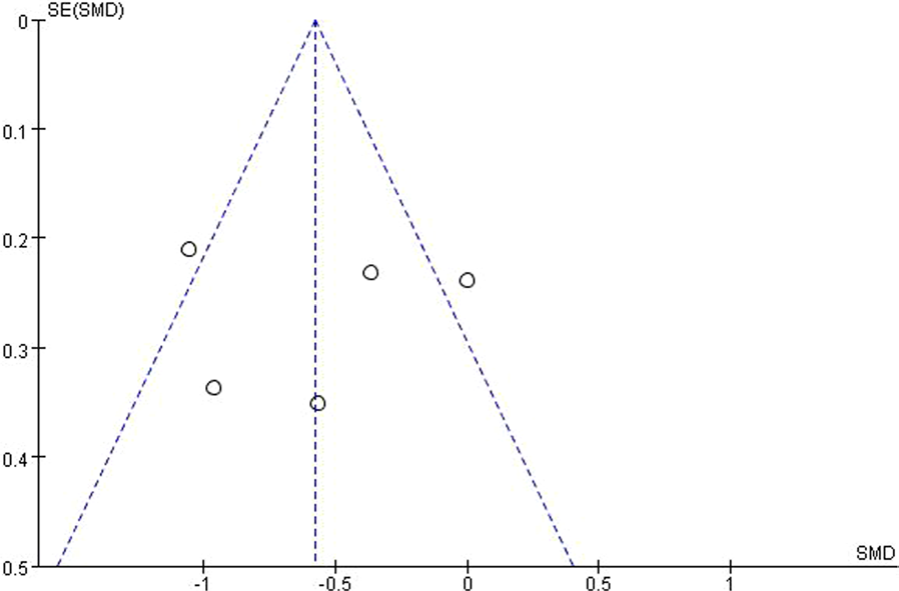
Funnel plot of studies involved in meta-analysis of JPS

We analyzed the publication bias of the literature studies with the selected outcome index of joint position sense. The results showed no significant publication bias (Fig. 4).

A sensitivity analysis of the results of the meta-analysis of JPS is performed by removing references one by one. We found that when removing the study of Pazit Levinger, the heterogeneity changed from 69% to 45%, and when the other studies were removed, there was no significant change in heterogeneity.

**Fig. 5:**
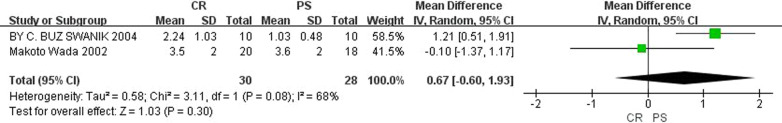
Proprioception of CR and PS prostheses

Two papers of five assessed subjects' proprioception by JPS compared CR-TKA (*n* = 30) and PS-TKA patients (*n* = 28). No significant differences in the JPS were identified between PS and CR groups (*P* = 0.30; *I*^2^ = 68%) (Fig. 5).

Only two studies discussed TTDPM in patients with TKA. One of them used healthy people and the contralateral as controls, the other compared joint movement sense before and after surgery. The sample size of the two articles was small, and 35 subjects were involved. The heterogeneity between the two studies was as high as 85% (*P* = 0.009). There was no significant difference in TTDPM between the experimental group and the control group (*P* = 0.48).

### Effects of TKA on balance

**Fig. 6:**
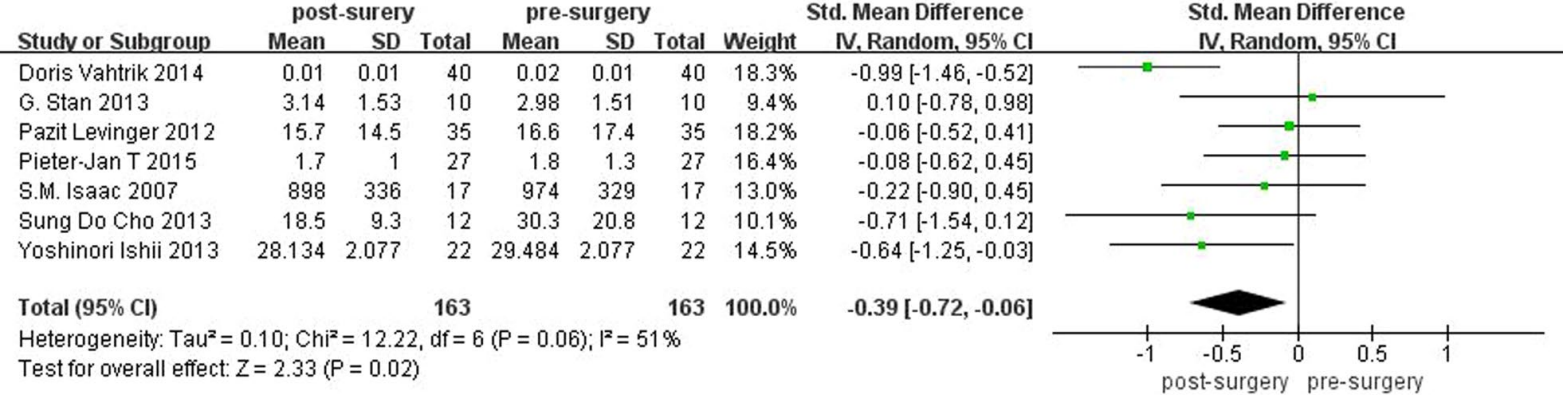
Meta-analysis of balance before and after surgery

Seven studies compared subjects' balance before and after surgery (*n* = 163) using the path of COP. There was significant heterogeneity among the studies, so random effects model analysis was used in the analysis of balance. The pooled standard mean difference of displacement of COP was − 0.39 (95% CI − 0.72 to − 0.06; *P* = 0.02; *I*^2^ = 51%). The results of static balance are shown in Fig. 6. It indicated that KOA patients had better static balance after TKA surgery than before surgery.

**Fig. 7:**
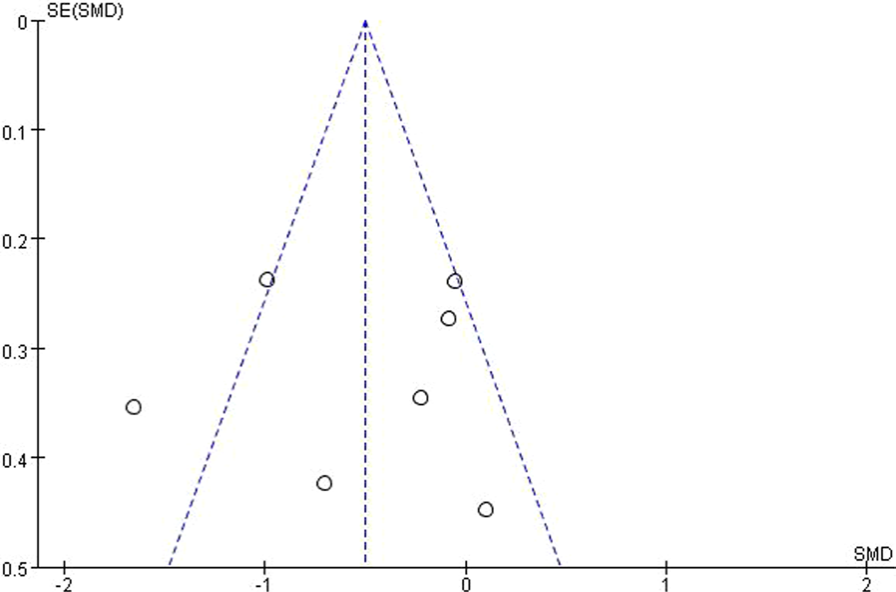
Funnel plot of studies involved in meta-analysis of balance

We analyzed the publication bias of the articles selected in the meta-analysis of balance, and the results showed no significant publication bias (Fig. 7).

**Fig. 8:**
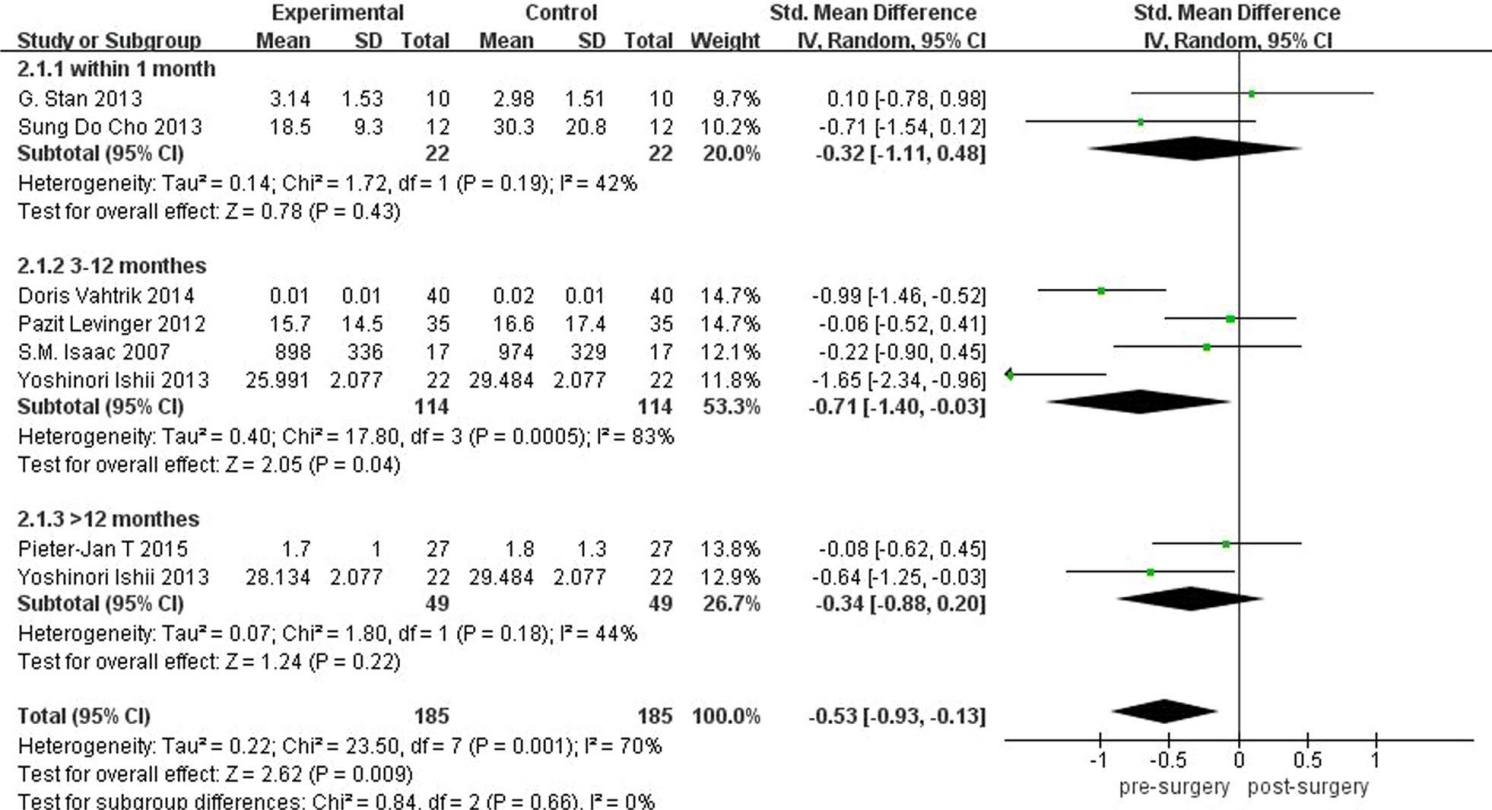
Balance subgroup analysis based on follow-up time

Taking into account the effect that different follow-up times may have on balance test results, we performed a subgroup analysis of preoperative and postoperative balance test results based on the duration of follow-up. Follow-up time of the three groups was 1 month after surgery, 3–12 months after surgery, and more than 12 months after surgery, respectively. The results are shown in Fig. 8. Twenty-two subjects in two studies were followed up in 1 month after surgery, and the pooled displacement of COP was − 0.32 (95% CI − 1.11 to 0.48; *P* = 0.43; *I*^2^ = 42%), which indicated that there was no significant difference in balance within 1 month after surgery. One hundred and fourteen subjects in four studies were followed up 3–12 months after surgery. The pooled displacement of COP was − 0.71 (95% CI − 1.40 to − 0.03; *P* = 0.04; *I*^2^ = 83%). In two studies, 49 subjects were followed for more than 1 year. The pooled displacement of COP was − 0.34 (95% CI − 0.88 to 0.20; *P* = 0.22; *I*^2^ = 44%).

We conducted a sensitivity analysis of the results of the meta-analysis of balance by removing references one by one. We found that the heterogeneity changed from 51 to 0% when removing the study of Doris Vahtrik.

## Discussion

This review examined the effect of TKA on knee proprioception, in terms of joint position sense, joint movement sense, and balance.

TKA has been a mature approach in treating end-stage knee osteoarthritis, relieving pain of KOA patients. With the development of rehabilitation medicine, many scholars have found that the deficiency of proprioceptive sensation and balance ability after operation is also a factor that cannot be ignored. However, no consistent conclusion has been drawn on the effect of TKA on proprioception. This review examined the effect of TKA on knee proprioception in terms of joint position sense and joint movement sense. The results cautiously indicated significantly better proprioception, in terms of JPS acuity after TKA compared to before TKA.

A sensitivity analysis of the results of the meta-analysis of JPS suggested that Pazit Levinger's study was a major source of heterogeneity in JPS analysis. In other studies, the mean angle of error represented the angle error of unilateral limb resetting a target angle. While in Pazit Levinger's study, the mean angle of error referred to the difference between bilateral limbs. The discrepancy in statistical and functional significance of the proprioceptive differences might be because the measurement techniques were insufficiently accurate. However, no selected study included sufficient information on the psychometrics of the measurement techniques. Therefore, the differences in reliability statistics between different JPS equipment and techniques could not be established. Furthermore, the time of follow-up ranged from 6 weeks to 12 months. It is believed that proprioception began to recover at 3 months after surgery, so the recovery degree of proprioception varied with the follow-up time.

It is thought that mechanoreceptors in the articular cartilage, ligaments, and muscles provide afferent information on the relative position and movement of the knee joint, and knee osteoarthritis impairs proprioception by disturbing transmission of this sensory information. Some suggested that proprioception was reduced since the surgical removed some tissues, while others hold opposite view. They believed the proprioception would be better after TKA since the surgery improved patients’ function and level of activity. Our results gave some support to this belief that TKA improved joint position sense of KOA patients. The mechanism for restoring joint sensation after TKA most likely involves the elimination of several deleterious factors in elderly and KOA patients [10, 30–32]. These patients have diminished joint sensation that can be linked to a loss of mechanoreceptors, pain, inflammation, laxity, decreased joint space, and physical activity levels [12, 32–37]. However, following TKA, the joint space and soft tissue tension have been reestablished, pain and chronic inflammation are reduced, and activities of daily living can be resumed. These changes may modify the response characteristics of mechanoreceptors in both capsuloligamentous and musculotendinous structures, enhancing the perception of joint position [16, 38].

Only two studies included assessing proprioception by TTDPM. One study used the preoperative as a control, while the other used healthy people as controls. The research of Géza Pap [20] showed that the postoperative TTDPM results of TKA patients were significantly different from those of the healthy control group, while Swanik [27] found no significant improvement between pre- and post-surgery.

Some believed that posterior cruciate ligament had different types of mechanoreceptors that detect joint position and joint motion [39]. The different design (CR-TKA or PS-TKA) of the prosthesis determined the retention of the posterior cruciate ligament. Some have suggested that preserving the posterior cruciate ligament for its neurosensory qualities might improve the outcome of TKA [27, 28]. However, whether retaining the posterior cruciate ligament enhanced joint proprioception or not was inconclusive. Our result showed that no significant differences in the JPS were identified between PS and CR groups, which might support the view that muscle rather than ligaments provides the primary afferent information in the sensorimotor system [40]. However, the sample size in the two papers was too small; more data are needed to confirm whether the two prostheses have different effects on the proprioception of patients after surgery.

Balance depends on visual and vestibular system, proprioception, and response of muscles. Some studies suggested that proprioception could be assessed indirectly through postural control. In terms of balance, we found significant improvement on balance after TKA, which was shown by the smaller displacement of COP sway.

A sensitivity analysis of the results of the meta-analysis of balance is performed by removing references one by one. We found that the heterogeneity changed from 51% to 0% when removing the study of Doris Vahtrik [28], which indicated that the study of Doris Vahtrik was the main source of heterogeneity. In this article, subjects were asked to stand with the right and left leg on different platforms, while the other studies used just one force plates. The difference between methods might be the source of heterogeneity. The same as JPS, 7 studies included in meta-analysis of balance used different techniques; the accuracy might be different among instruments.

The results of balance subgroup analysis based on follow-up time showed that there is no significant difference in balance within 1 month after surgery. This may be because the time after surgery is too short and balance performance has not been fully restored. Great improvement on balance was shown in TKA patients 3–12 months after surgery. This was consistent with the overall analysis results, indicating that the balance ability gradually recovered after TKA, and significant improvement in balance did not occur until 3 months after surgery. In two studies, 49 subjects were followed for more than 1 year, which suggested that there was no significant difference in balance over a year after surgery. That could be because fewer studies followed for longer than a year, and the sample size was small. However, our subgroup analysis demonstrated that balance did not recover until 3 months after surgery.

There are well-established and recognized methods for measuring joint motion and joint position sense while joint force sense has been less studied. Joint force sense is often performed at the standing/functional position and an indispensable part to reflect the proprioceptive efferent movement ability and plays a crucial role in maintaining joint stability and postural balance. Few existing studies that included joint force test adopted the method of allowing subjects to reproduce the target force. In 1993, Kyberd [41] measured the reflex contraction latency of hamstring in 30 patients with anterior cruciate ligament injury using a self-designed device, which included a motion sensor, an EMG sensor, and a pneumatic device to synchronize the motion sensor and the EMG sensor. The motion sensor was placed on the anterior of the proximal tibia, and the EMG sensor was placed on the hamstring. The two sensors recorded the moment when the proximal tibia began to lean forward under stress and the moment when the hamstring began to generate EMG activation in the flexion motion of the knee, respectively. The time difference between the two moments was considered as the reflex contraction latency, which provided an indirect measure of the proprioception ability of knee joint. However, this test method is highly subjective, and subjects are easily disturbed by environmental factors, so it is difficult to obtain objective and accurate test results. The new method of measuring the knee force sense is worth paying attention, which will make progress with knee proprioception on TKA patients. At the same time, the heterogeneity of the methods among the studies made it hard to draw the specific conclusions on how knee proprioception of KOA patients will change after TKA, which is a limitation of this study.

## Conclusions

To conclude, no standardized comprehensive evaluation protocol presently exists though different assessment tools are available to measure proprioception. Contrasting results were found in the literature since some studies found that TKA improved proprioception in KOA patients, while others found no difference in proprioception. These differences were seen whether the proprioception was assessed by JPS, or it was indirectly assessed by static balance. However, the lack of sufficient data on the TTDPM and dynamic balance made it difficult to draw a conclusion about whether or not the sense of motion improved after surgery. In addition, the method for measuring and evaluating knee joint force sense is worth paying attention, which will make progress with knee proprioception on TKA patients.

## Abbreviations

KOA: Knee osteoarthritis
TKA: Total knee arthroplasty
COP: Center of pressure
JPS: Joint position sense
TTDPM: Threshold to detect passive movement
WMD: Weighted mean difference
SMD: Standard mean difference
